# Predictive value of circulating inflammatory biomarkers for early-onset post-stroke cognitive impairment: a prospective cohort study

**DOI:** 10.3389/fneur.2025.1565613

**Published:** 2025-04-24

**Authors:** Weiquan Huang, Libin Liao, Qian Liu, Rongchao Ma, Wentong Hu, Yuan Dai, Luna Wang, Dujuan Sha

**Affiliations:** ^1^Department of General Practice, Nanjing Drum Tower Hospital Clinical College of Nanjing Medical University, Nanjing, Jiangsu, China; ^2^Department of General Practice, Nanjing Drum Tower Hospital Clinical College of Xuzhou Medical University, Nanjing, China; ^3^Department of General Practice, Nanjing Drum Tower Hospital, Affiliated Hospital of Medical School, Nanjing University, Nanjing, China; ^4^Institute of Functional Biomolecules, State Key Laboratory of Pharmaceutical Biotechnology, Nanjing University, Nanjing, China

**Keywords:** stroke, post-stroke cognitive impairment, early-onset, inflammation, circulating biomarkers

## Abstract

**Introduction:**

Stroke ranks as the second leading cause of mortality and the third leading cause of disability globally. Post-stroke cognitive impairment (PSCI) is a prevalent complication following acute ischemic stroke, imposing substantial burdens on patients, families, and society. This study aimed to explore the potential of circulating immune-inflammatory markers as predictors of PSCI.

**Methods:**

Conducted as a prospective observational cohort study from June 2023 to August 2024 at the Affiliated Drum Tower Hospital, Medical School of Nanjing University, it included patients experiencing their first acute ischemic stroke within 72 h of symptom onset. Cognitive assessments were conducted 7 to 10 days post-stroke using the Montreal Cognitive Assessment (MoCA), with scores below 23 indicating PSCI.

**Results:**

A total of 146 patients meeting the inclusion criteria were recruited, with 71 patients exhibiting PSCI during the acute phase of stroke. Compared to patients in the post-stroke no cognitive impairment (PSNCI) group, those with PSCI demonstrated significantly elevated peripheral blood neutrophil-to-lymphocyte ratio (NLR), globulin-to-lymphocyte ratio (GLR), and C-reactive protein-to-lymphocyte ratio (CLR), while the lymphocyte-to-monocyte ratio (LMR) was notably reduced (all *p* < 0.05). Both univariate and multivariate logistic regression analyses identified GLR as independently associated with PSCI. After adjusting for common clinical variables, the odds ratio (OR) for the highest tertile of GLR compared to the lowest was 6.20 (95% CI, 2.10–18.33; *p* = 0.001). The optimal GLR cutoff was 18.22, with a sensitivity of 62.0%, specificity of 78.7%, and an area under curve (AUC) of 0.726.

**Conclusion:**

This study indicates that elevated circulating GLR levels during the acute phase post-stroke onset are an independent risk factor for early-onset PSCI, even after adjusting for clinically relevant variables.

## 1 Introduction

Stroke is the second leading cause of death and the third leading cause of disability in the world (1). Depending on the etiology and pathogenesis, stroke can be classified as ischemic stroke or hemorrhagic stroke. Of these, ischemic stroke is the most common type, accounting for approximately 87% of all stroke patients (2). Post-stroke cognitive impairment (PSCI) is one of the most common complications of acute ischemic stroke, with a prevalence ranging from 15% to 70%, depending on the clinical features and stroke severity (3), which imposes a heavy burden on the patient, family, and society. PSCI includes cognitive impairment that occurs within 3 to 6 months after stroke and is clinically characterized by persistent impairment in one or more core cognitive domains, such as attention, memory, executive function, language, and visuospatial ability, and is associated with a significantly increased risk of death, disability, and depression up to 5 years after the onset of the stroke (4). Common risk factors for PSCI are advanced age, low education, hypertension, diabetes mellitus, atrial fibrillation, smoking, family history, sedentary lifestyle, stroke subtype, and stroke severity (5–7).

The diagnosis of PSCI is usually based on clinical assessment, neuropsychological evaluation, and neuroimaging (8, 9). The Montreal Cognitive Assessment (MoCA) and the Mini-Mental State Examination (MMSE) are the most widely used cognitive tests for PSCI research. Factors such as the patient’s education degree, evaluation by different personnel, and the selection of different scales can affect the assessment results; these two tools are mainly applied to neurodegenerative diseases and have low sensitivity in detecting PSCI, which can lead to the underdiagnosis of PSCI (10). Computer tomography (CT) remains the standard imaging modality in clinical practice due to its advantages over magnetic resonance imaging (MRI) in terms of speed, cost, and fewer contraindications (11). MRI brain scans are generally more helpful in dementia assessment and are considered the “gold standard” for neuroimaging-based assessment (12). However, if neuroimaging is to be combined with clinical characterization as the primary diagnostic support for PSCI, it is essential to have experienced imaging physicians and to perform high-quality scans to obtain accurate information.

Therefore, blood biomarkers hold promising potential due to their availability, low invasiveness, objectivity, and cost-effectiveness. An increasing number of research studies have shown that blood biomarkers play an important role in the occurrence and development of PSCI. There are currently no precise blood biomarkers for PSCI risk prediction and early diagnosis at home and abroad. Acute cerebral ischemia releases damage-associated molecular patterns that trigger brain intrinsic immune cells (microglia) and recruit peripheral innate immune cells, including neutrophils and monocytes/macrophages for infiltration, leading to exacerbated ischemic injury (13). Meanwhile, cerebral ischemia releases harmful substances, especially necrotic cellular debris, which triggers an inflammatory cascade in the immune system, leading to subsequent repair processes and tissue damage (14). Imbalanced expression of pro- and anti-inflammatory cytokines in the brain activates cerebral microvessels and disrupts the blood–brain barrier, exacerbating neurological dysfunction and ultimately leading to cognitive impairment and dementia (15, 16). Studies have shown a strong correlation between the immune response in the acute phase of stroke and long-term cognitive function (17). A recent study found that the neutrophil-to-lymphocyte ratio (NLR) during the acute phase of ischemic stroke was independently correlated with PSCI at 3 months post-stroke (18), which is consistent with the findings of several other studies (19, 20). Therefore, the immune-inflammatory response plays a significant role in the pathogenic process of PSCI. Peripheral blood cell counts and biochemical indices are among the most accessible laboratory indices in the clinic. In this study, peripheral blood lymphocyte count was combined with neutrophil count, monocyte count, serum globulin, and C-reactive protein (CRP) to investigate the predictive value of the NLR, lymphocyte-to-monocyte ratio (LMR), globulin-to-lymphocyte ratio (GLR), and C-reactive protein-to-lymphocyte ratio (CLR) in early-onset PSCI.

## 2 Materials and methods

### 2.1 Patients

**Figure 1:**
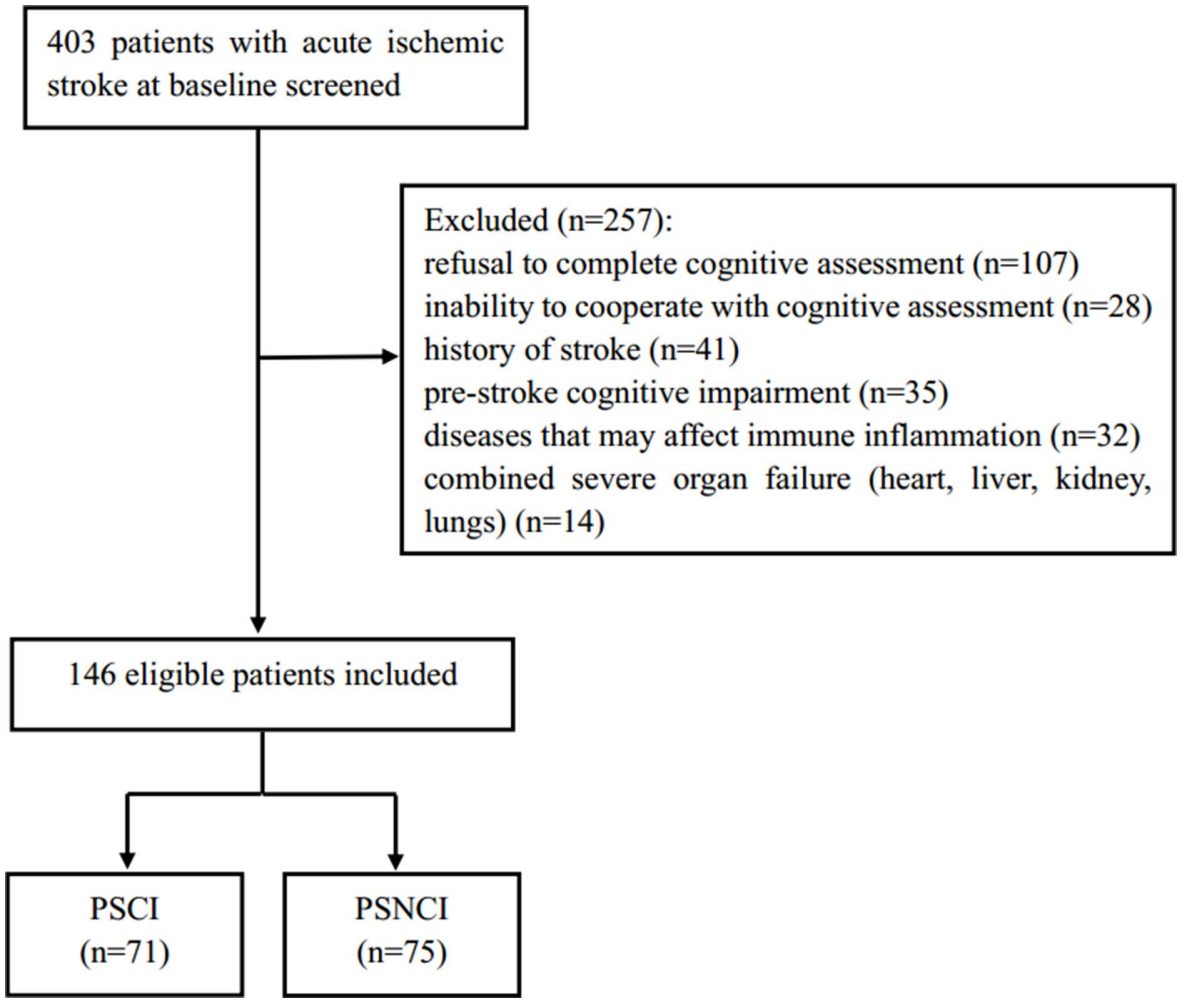
Flow diagram.

This study is a prospective observational cohort study that consecutively included 146 patients with acute ischemic stroke, admitted to Nanjing Drum Tower Hospital, affiliated with Nanjing University Medical School, from June 2023 to August 2024. The inclusion criteria were as follows: (1) age ≥ 18 years; (2) first ischemic stroke diagnosed by CT or MRI; (3) time from onset to admission within 72 h; (4) complete clinical information available; (5) consent obtained from the subjects and their families, with a signed informed consent form. The exclusion criteria were: (1) pre-stroke cognitive impairment or dementia (including Alzheimer’s disease, frontotemporal dementia, Parkinson’s disease dementia, Lewy body dementia, and mixed dementia); (2) inability to cooperate with cognitive function assessment (severe aphasia, hearing impairment, consciousness disorder, severe visual blurring, and writing disorder); (3) severe cardiac, hepatic, renal insufficiency, and respiratory failure; (4) diseases that may cause inflammation and immune responses (acute infection, tumors, hematological diseases, autoimmune diseases, recent major surgery history, and recent trauma history); (5) use of medications that may interfere with the inflammation and immune system (antibiotics, glucocorticoids, immunosuppressants, and non-steroidal anti-inflammatory drugs); (6) refusal to complete cognitive assessment; and (7) history of stroke. Based on MoCA scores, patients were divided into a PSCI group (71 cases) and a post-stroke no cognitive impairment (PSNCI) group (75 cases). Figure 1 shows the detailed flowchart. This study adheres to the Declaration of Helsinki of the World Medical Association and was approved by the Ethics Committee of Nanjing Drum Tower Hospital (Protocol Number: 2022-333-01). The procedures comply with national and institutional guidelines.

### 2.2 Baseline data collection

Demographic characteristics (gender, age, and education level), medical history (hypertension, diabetes mellitus, coronary artery disease, atrial fibrillation, smoking, and alcohol consumption), clinical information (systolic and diastolic blood pressures), and laboratory findings (peripheral blood cell counts, lipid levels, fasting plasma glucose (FPG), CRP, and globulin levels) were collected after admission. Hypertension was defined as a history of hypertension or the use of anti-hypertensive medications. Diabetes mellitus was defined as a history of diabetes or the use of hypoglycemic medications. Smoking history was defined as smoking ≥1 cigarette per day for >6 months. Alcohol consumption history was defined as consuming ≥1 drink per week, with each drink containing ≥50 mL of alcohol for >6 months. Baseline stroke severity was assessed by an experienced clinician using the National Institutes of Health Stroke Scale (NIHSS), and baseline disability level was assessed using the Barthel Index (BI) (21, 22). Fasting blood samples were collected from all patients the morning after admission. Lipids, FPG, and globulin were measured using a biochemistry analyzer (Beckman, AU5421, United States); glycated hemoglobin (HbA1c) was measured using a glycated hemoglobin analyzer (Tosoh, G8, Japan); and blood counts were measured using a fully automated hemocytometer analyzer (Sysmex Corporation, XE-5000, Japan), with NLR, LMR, GLR, and CLR calculated. The NLR is calculated by dividing the neutrophil count by the lymphocyte count. Using the same methodology, the LMR, GLR, and CLR can be derived.

### 2.3 Assessment of cognitive function

The study’s outcome was PSCI within 7–10 days after stroke. To minimize potential bias associated with the subjectivity of the MoCA, cognitive assessment was performed using the MoCA scale by a clinician with specialized training who was blinded to the clinical and laboratory data. The domain of cognitive assessment encompasses several key components, including visuospatial abilities and executive function (5 points), naming (3 points), attention (6 points), language (3 points), abstraction (2 points), delayed recall (5 points), and orientation (6 points). The total MoCA score was 30 points, and for patients with fewer than 12 years of education, 1 point was added to the total MoCA score (if <30 points) to correct for education effects. For this study, a MoCA score of less than 23 was defined as cognitive impairment (23). The MoCA test takes approximately 10–15 min. Standardized instructions were provided in the subjects’ native language using a validated translated version of the MoCA. Subjects had access to necessary assistive devices such as hearing aids and eyeglasses.

### 2.4 Statistical analysis

All statistical tests were performed in SPSS 25.0, and statistical graphs were plotted using GraphPad Prism 10.3.0. The Kolmogorov–Smirnov test was used to evaluate the normality of the quantitative data. Measurements that conformed to normal distribution were expressed as mean and standard deviation, and comparisons between the two groups were made using the independent samples t-test. Measurement information that did not conform to normal distribution was expressed as a median and interquartile range, and comparisons between two groups were made using the independent samples Mann–Whitney U-test. Count data were expressed as frequencies and percentages, and comparisons between groups were made using the chi-square test. Spearman’s correlation coefficients were used to correlate MoCA scores with clinical baseline data. Univariable logistic regression analysis was conducted to investigate the association between baseline characteristics and PSCI, and all variables with a *p*-value of <0.1 were included in subsequent multivariable logistic regression models. The odds ratio (OR) or adjusted OR combined with the 95% confidence interval (CI) showed the presence of an association. The potential predictive role of GLR on PSCI was also assessed using receiver operating characteristic (ROC) curves. All statistical analyses were defined as statistically significant with a two-sided *p*-value of <0.05.

## 3 Results

### 3.1 Baseline characteristics

**Figure 2:**
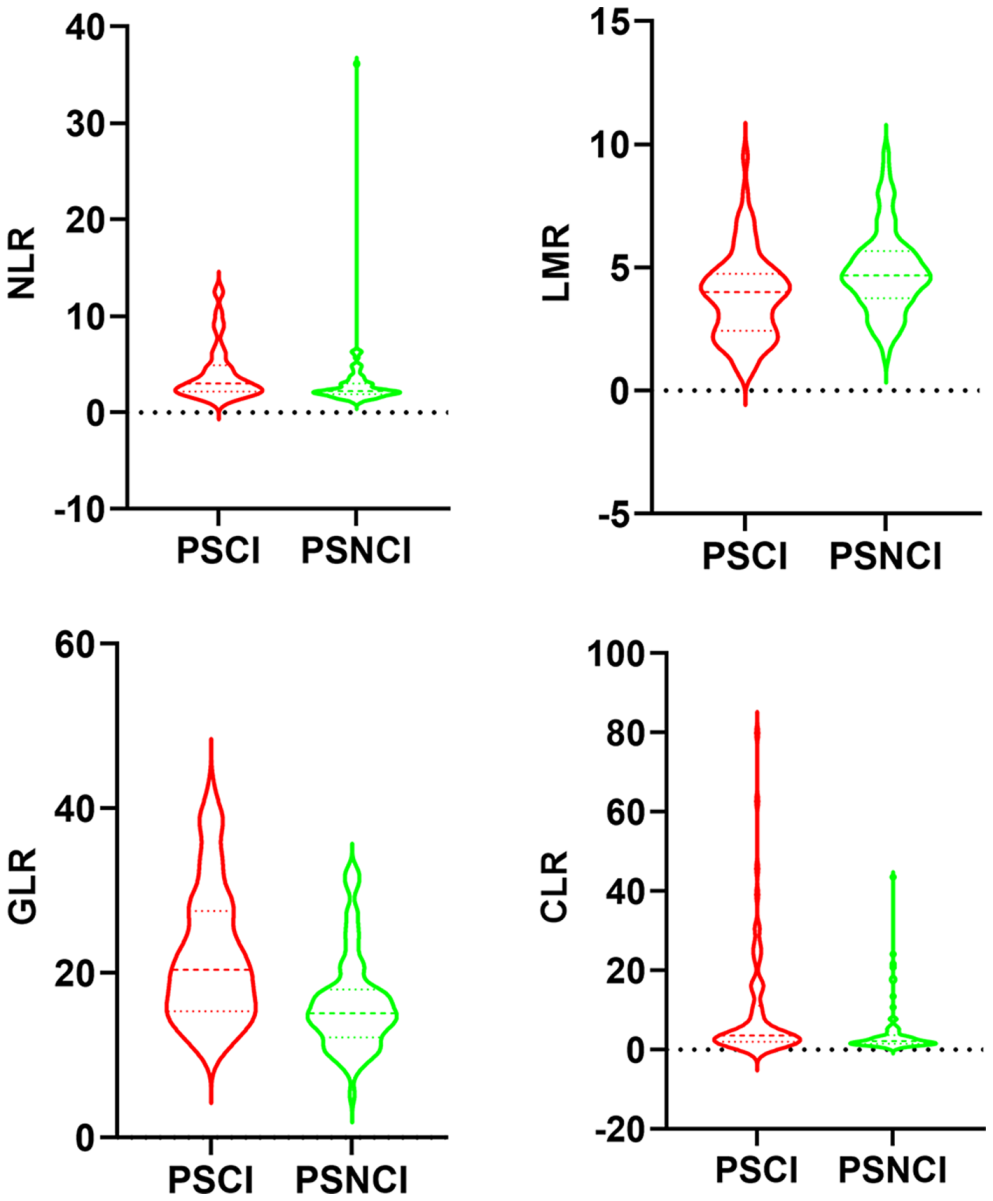
Violin plot of NLR, LMR, GLR, and CLR distribution in the PSCI and PSNCI subgroups.

Table 1 shows the differences in baseline characteristics between the PSCI and PSNCI groups. In this study, a total of 146 patients with first ischemic stroke who met the inclusion criteria were identified, including 99 males and 47 females, with ages ranging from 33 to 88 years and a mean age of 64.4 ± 12.6 years. In the acute phase of stroke, 71 (48.6%) patients presented with PSCI. Compared to the PSNCI group, patients in the PSCI group were older, had fewer years of education, lower BI scores, and higher NIHSS scores. There were no significant differences in sex ratio, history (hypertension, diabetes, coronary artery disease, atrial fibrillation, smoking, and alcohol consumption), or systolic and diastolic blood pressure between the two groups. In terms of laboratory tests, peripheral NLR, GLR, and CLR levels were significantly higher in the PSCI group than in the PSNCI group (All *p* < 0.05). Hemoglobin (Hb), total cholesterol (TC), low-density lipoprotein cholesterol (LDL-C), and LMR levels were significantly lower in patients in the PSCI group than in those in the PSNCI group (all *p* < 0.05). The two groups had no significant differences in leukocyte count, platelet count, CRP, triglyceride (TG), high-density lipoprotein cholesterol (HDL-C), FPG, and HbA1c. Figure 2 shows each subgroup’s violin plots of NLR, LMR, GLR, and CLR distribution.

**Table 1:** Baseline characteristics between the PSCI group and PSNCI group.

Baseline characteristics	Total (*n* = 146)	PSCI (*n* = 71)	PSNCI (*n* = 75)	*p*
Demographics				
Male, *n* (%)	99 (67.8)	43 (60.6)	56 (74.7)	0.068
Age, mean ± SD (years)	64.4 ± 12.6	66.7 ± 12.0	62.2 ± 12.7	0.03^*^
Education level, median (IQR) (years)	9.0 (6.0,12.0)	9.0 (6.0,9.0)	12.0 (9.0,12.0)	<0.001^***^
Medical history, *n* (%)				
Hypertension	100 (68.5)	52 (73.2)	48 (64.0)	0.230
Diabetes mellitus	45 (30.8)	20 (28.2)	25 (33.3)	0.499
Coronary artery disease	14 (9.6)	10 (14.1)	4 (5.3)	0.073
Atrial fibrillation	11 (7.5)	8 (11.3)	3 (4.0)	0.096
Smoking	48 (32.9)	18 (25.4)	30 (40.0)	0.060
Alcohol consumption	24 (16.4)	11 (15.5)	13 (17.3)	0.764
Clinical characteristics				
BI score	90 (55,100)	80 (45,95)	95 (75,100)	<0.001^***^
NIHSS score	3 (2,6)	4 (2,7)	3 (1,4)	0.001^**^
MoCA score	23 (19,26)	19 (14,21)	26 (24,28)	<0.001^***^
SBP, mean ± SD (mmHg)	145.9 ± 20.8	143.8 ± 21.9	147.8 ± 19.8	0.240
DBP, mean ± SD (mmHg)	84.6 ± 12.9	83.0 ± 14.3	86.1 ± 11.3	0.145
Laboratory characteristics				
Leukocyte, median (IQR) (10^9^/L)	6.5 (5.4,8.3)	6.5 (5.4,8.7)	6.5 (5.4,7.8)	0.820
Hb, median (IQR) (g/L)	135.0 (125.0,147.0)	132.0 (123.0,145.0)	141.0 (126.0,150.0)	0.030^*^
Platelet, median (IQR) (10^9^/L)	201.5 (157.8,239.3)	194.0 (154.0,239.0)	202.0 (168.0,240.0)	0.422
TG, median (IQR) (mmol/L)	1.3 (1.0,1.9)	1.2 (0.9,1.7)	1.5 (1.0,2.1)	0.069
TC, median (IQR) (mmol/L)	4.5 (3.5,5.3)	4.1 (3.3,4.9)	4.6 (3.7,5.4)	0.018^*^
HDL-C, median (IQR) (mmol/L)	1.1 (0.9,1.3)	1.1 (0.9,1.3)	1.2 (0.9,1.4)	0.236
LDL-C, median (IQR) (mmol/L)	2.5 (1.9,3.3)	2.3 (1.8,3.0)	2.8 (2.1,3.4)	0.022^*^
FPG, median (IQR) (mmol/L)	5.2 (4.6,6.5)	5.5 (4.6,6.4)	5.2 (4.5,6.7)	0.541
HbA1c, median (IQR) (%)	6.0 (5.5,6.6)	6.0 (5.6,6.7)	5.9 (5.4,6.6)	0.558
NLR, median (IQR)	2.5 (2.0,3.8)	3.0 (2.2,4.9)	2.2 (1.9,3.0)	0.001^**^
LMR, median (IQR)	4.3 (3.0,5.3)	4.0 (2.4,4.8)	4.7 (3.8,5.7)	<0.001^***^
GLR, median (IQR)	17.1 (13.8,22.8)	20.4 (15.4,27.6)	15.1 (12.2,18.0)	<0.001^***^
CLR, median (IQR)	2.9 (1.7,5.8)	3.6 (2.1,11.1)	2.2 (1.5,3.7)	0.002^**^

PSCI, post-stroke cognitive impairment; PSNCI, post-stroke no cognitive impairment; SD, standard deviation; IQR, interquartile range; BI, Barthel Index; NIHSS, National Institute of Health Stroke Scale; MoCA, Montreal Cognitive Assessment; SBP, systolic blood pressure; DBP, diastolic blood pressure; Hb, hemoglobin; TG, triglyceride; TC, total cholesterol; HDL-C, high-density lipoprotein cholesterol; LDL-C, low-density lipoprotein cholesterol; FPG, fasting plasma glucose; HbA1c, glycosylated hemoglobin; NLR, neutrophil-to-lymphocyte ratio; LMR, lymphocyte-to-monocyte ratio; GLR, globulin-to-lymphocyte ratio; CLR, C-reactive protein-to-lymphocyte ratio. ^*^p < 0.05, ^**^p < 0.01, ^***^p < 0.001.

### 3.2 Correlation analysis of NLR, LMR, GLR, and CLR with MoCA

**Figure 3:**
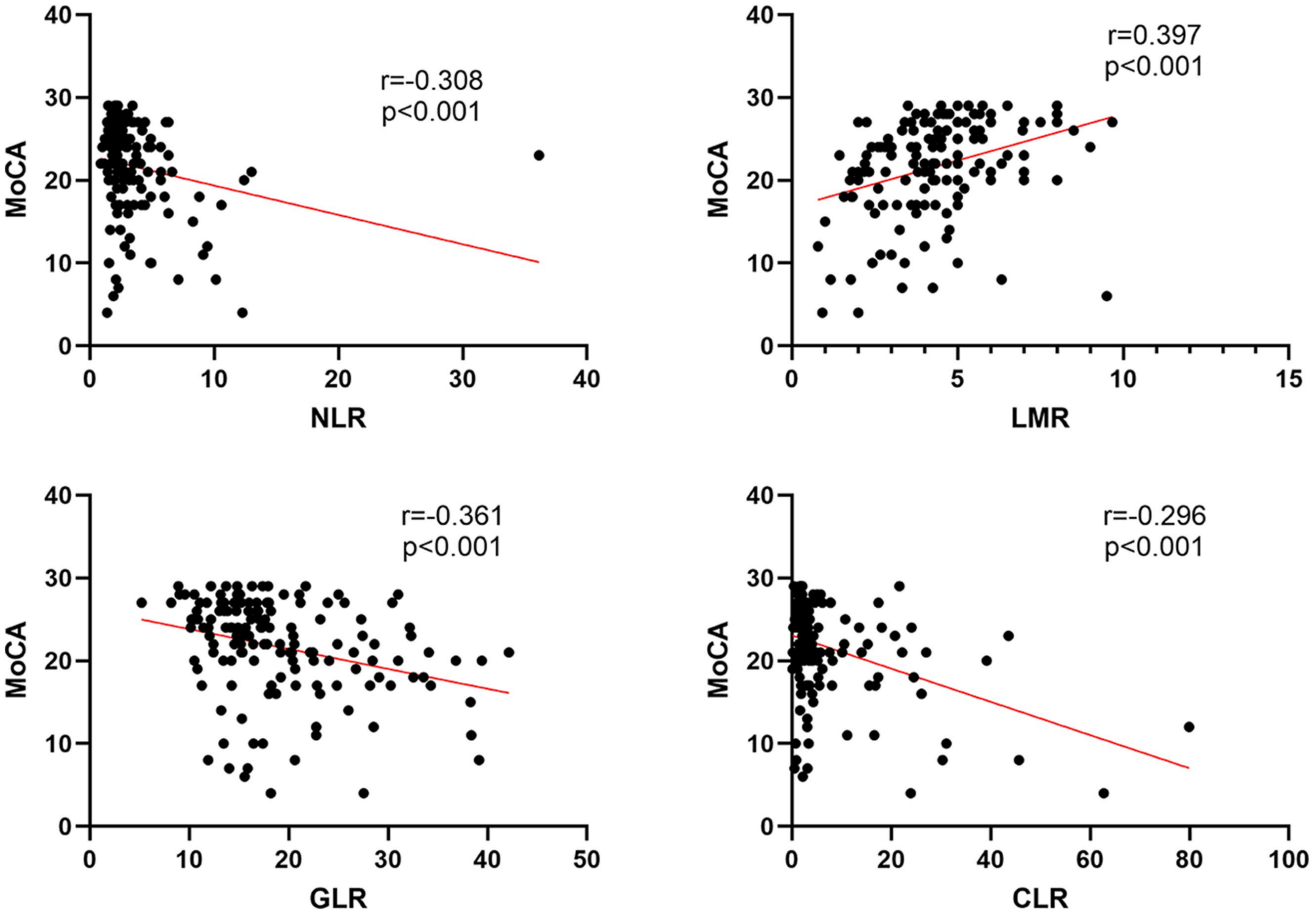
Spearman’s correlation analysis of NLR, LMR, GLR, and CLR with MoCA.

Figure 3 displays the results of Spearman’s correlation analysis of NLR, LMR, GLR, and CLR with MoCA. The results showed that LMR (r = 0.397, *p* < 0.001) was positively correlated with MoCA score, while NLR (r = −0.308, *p* < 0.001), GLR (r = −0.361, *p* < 0.001), and CLR (r = −0.296, *p* < 0.001) were negatively correlated with MoCA score.

### 3.3 Logistic regression analysis of the relationship between various indicators and PSCI

The univariate logistic regression analysis revealed that age, education level, BI score, NIHSS score, Hb, TC, LDL-C, LMR, GLR, and CLR were significantly associated with PSCI (all *p* < 0.05). After correcting for variables with a *p*-value of <0.1 in the univariate logistic regression analysis, the multivariate logistic regression analysis revealed that education level (OR, 0.78; 95% CI, 0.68–0.90; *p* < 0.001) and GLR (OR, 1.11; 95% CI, 1.03–1.19; *p* = 0.008) were independently associated with PSCI (Table 2).

**Table 2:** Univariable and multivariable logistic regression analyses for baseline data.

Baseline characteristics	Univariable analysis		Multivariable analysis	
	OR (95%CI)	*p*	Adjusted OR (95%CI)	*p*
Demographics				
Sex, male	0.52 (0.26,1.06)	0.070	0.56 (0.18,1.74)	0.319
Age	1.03 (1.00,1.06)	0.032^*^	1.01 (0.97,1.05)	0.781
Education level	0.79 (0.71,0.88)	<0.001^***^	0.78 (0.68,0.90)	<0.001^***^
Clinical characteristics				
Hypertension	1.54 (0.76,3.12)	0.231		
Diabetes mellitus	0.78 (0.39,1.59)	0.500		
Coronary artery disease	2.91 (0.87,9.75)	0.083	4.61 (0.94,22.67)	0.060
Atrial fibrillation	3.05 (0.78,11.99)	0.111		
Smoking	0.51 (0.25,1.03)	0.061	1.01 (0.36,2.86)	0.988
Alcohol consumption	0.87 (0.36,2.10)	0.764		
Clinical characteristics				
BI score	0.97 (0.96,0.99)	<0.001^***^	0.98 (0.96,1.01)	0.111
NIHSS score	1.21 (1.08,1.35)	0.001^**^	0.98 (0.80,1.20)	0.840
SBP	0.99 (0.98,1.01)	0.240		
DBP	0.98 (0.96,1.01)	0.146		
Laboratory characteristics				
Leukocyte	0.99 (0.92,1.07)	0.871		
Hb	0.98 (0.96,1.00)	0.029^*^	1.00 (0.97,1.03)	0.935
Platelet	1.00 (0.99,1.01)	0.852		
TG	0.73 (0.50,1.06)	0.094	0.90 (0.50,1.62)	0.727
TC	0.71 (0.53,0.94)	0.018^*^	0.40 (0.08,2.00)	0.267
HDL-C	0.62 (0.22,1.74)	0.363		
LDL-C	0.67 (0.47,0.96)	0.029^*^	2.77 (0.39,19.67)	0.308
FPG	1.05 (0.89,1.24)	0.563		
HbA1c	0.99 (0.81,1.21)	0.909		
NLR	1.11 (0.97,1.27)	0.135		
LMR	0.71 (0.57,0.87)	0.001^**^	1.08 (0.79,1.47)	0.639
GLR	1.13 (1.07,1.20)	<0.001^***^	1.11 (1.03,1.19)	0.008^**^
CLR	1.06 (1.01,1.11)	0.012^*^	1.03 (0.98,1.09)	0.261

OR, odds ratio; CI, confidence interval; BI, Barthel Index; NIHSS, National Institute of Health Stroke Scale; SBP, systolic blood pressure; DBP, diastolic blood pressure; Hb, hemoglobin; TG, triglyceride; TC, total cholesterol; HDL-C, high-density lipoprotein cholesterol; LDL-C, low-density lipoprotein cholesterol; FPG, fasting plasma glucose; HbA1c, glycosylated hemoglobin; NLR, neutrophil-to-lymphocyte ratio; LMR, lymphocyte-to-monocyte ratio; GLR, globulin-to-lymphocyte ratio; CLR, C-reactive protein-to-lymphocyte ratio. ^*^p < 0.05, ^**^p < 0.01, ^***^p < 0.001.

### 3.4 Predicting the risk of PSCI based on GLR tertiles

The tertile levels of GLR were as follows: tertile 1 (GLR ≤ 14.89) (*n* = 49), tertile 2 (14.89 < GLR ≤ 20.46) (*n* = 49), and tertile 3 (GLR > 20.46) (*n* = 48). Compared to the lowest tertile, logistic regression analysis unadjusted for clinical variables showed an OR of 7.46 (95% CI, 3.04–18.31; *p* < 0.001) for the highest tertile of GLR. The same result was still obtained after stepwise adjustment for important variables such as age, sex, education level, NIHSS score, hypertension, diabetes, coronary heart disease, atrial fibrillation, history of smoking and alcohol consumption, TG, TC, HDL-C, LDL-C, Hb, and FBG (OR, 6.20; 95% CI, 2.10–18.33; *p* = 0.001) (Table 3).

**Table 3:** Risk of post-stroke cognitive impairment according to GLR tertiles.

Tertiles	Unadjusted	*p*	Model 1	*P*	Model 2	*p*	Model 3	*p*
	OR (95%CI)		OR (95%CI)		OR (95%CI)		OR (95%CI)	
T1 (≤14.89) (*n* = 49)	Reference		Reference		Reference		Reference	
T2 (14.89–20.46) (*n* = 49)	2.45 (1.05,5.71)	0.038	2.01 (0.80,5.05)	0.136	2.31 (0.86,6.21)	0.097	1.96 (0.69,5.54)	0.204
T3 (>20.46) (*n* = 48)	7.46 (3.04,18.31)	<0.001	5.99 (2.22,16.13)	<0.001	6.33 (2.21,18.13)	0.001	6.20 (2.10,18.33)	0.001
*p* for trend	<0.001		<0.001		0.001		0.001	

Model 1 adjusts for age, sex, and education level. Model 2 further adjusts for NIHSS score, history of hypertension, diabetes mellitus, coronary artery disease, atrial fibrillation, smoking, and alcohol consumption based on model 1. Model 3 further adjusts for TG, TC, HDL-C, LDL-C, Hb, and FBG based on model 2.

### 3.5 ROC analysis of GLR for predicting PSCI

**Figure 4:**
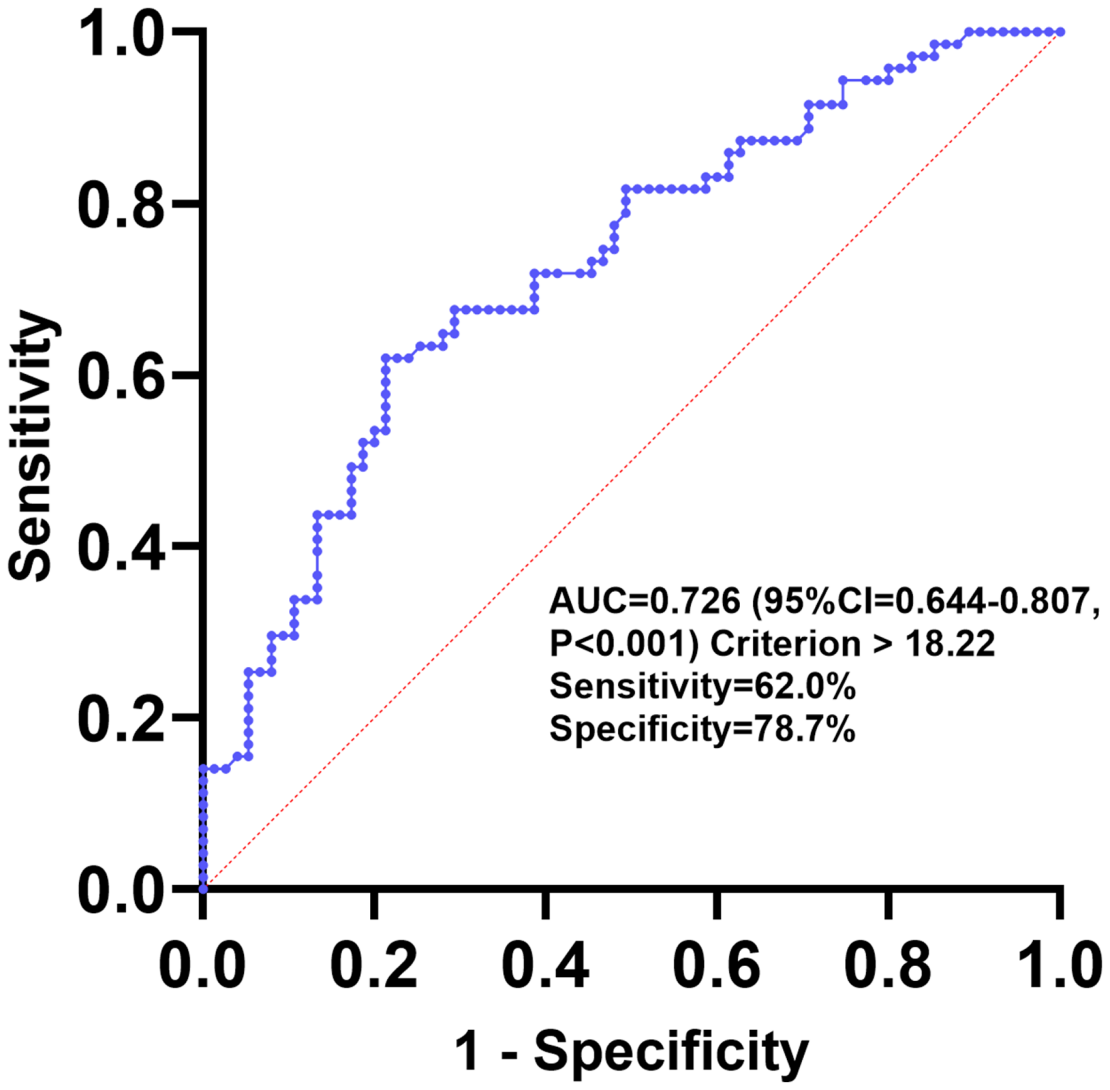
Receiver operating characteristic (ROC) curve for GLR as a predictor of PSCI.

The diagnostic value of GLR for PSCI was evaluated using ROC analysis, and the area under the curve (AUC) was 0.726 (95% CI, 0.644–0.807; *p* < 0.001) (Figure 4). The optimal cutoff value based on the Youden index was >18.22, with a sensitivity of 62.0% and a specificity of 78.7%.

## 4 Discussion

In the acute phase of stroke, more attention is typically given to limb motor, speech, and visual deficits, while cognitive dysfunction in the early phase of stroke is often overlooked. Studies have indicated that assessment of cognitive function within the first week after stroke predicts long-term cognitive outcomes, functional outcomes, and mortality (24). MoCA scores in the acute phase of stroke are associated with cognitive decline or the development of dementia within 6–9 months (25). Previous meta-analyses showed that PSCI has the highest incidence in the first few months following a stroke, affecting approximately 75% of stroke survivors during the acute phase (26). Therefore, assessing cognitive function in the acute phase of stroke is particularly important.

This research explored circulating biomarkers of cognitive impairment in the acute phase of stroke. Our study found that NLR, GLR, and CLR were significantly higher in the PSCI group, whereas LMR was lower than in the PSNCI group. The human immune system consists of two components: adaptive immunity (natural killer cells, B cells, and T cells) and innate immunity (macrophages, monocytes, neutrophils, mast cells, and dendritic cells) (27). Previous studies have demonstrated that hyperactivated neutrophils are associated with the progression of Alzheimer’s disease (AD), and higher neutrophil-to-lymphocyte ratios are associated with any dementia (28). Hyperactivity of innate immunity can mediate neuronal damage and death through microglia activation (29). A cohort study of 361,653 participants found that enhanced innate immunity was associated with an increased risk of dementia, and enhanced adaptive immunity was associated with a decreased risk of dementia (30). The results of another prospective cohort study showed that higher systemic immunoinflammatory index (SII) levels at admission in patients with acute ischemic stroke were independently associated with the development of PSCI 3 months later (31). Immune dysfunction occurs with aging, leading to chronic inflammation; chronic inflammation exacerbates neuronal damage and interferes with glial cell circuits, leading to cognitive impairment and dementia (32, 33). A meta-analysis has shown that sustained elevation of CRP in chronic inflammation is directly associated with an increased risk of developing AD later in life (34). CRP is a pentameric protein synthesized in the liver under the regulation of interleukin 6 (IL-6) and interleukin 1β (IL-1β), which plays a key role in both acute and chronic inflammatory responses (35). In our study, although there were significant differences in NLR, LMR, and CLR levels between the two groups (all *p* < 0.05), they were not found to be independently associated with PSCI in the multivariable logistic regression analysis. The possible explanation for this is that patients in the PSCI group had higher NIHSS scores and more severe strokes, which triggered a more severe inflammatory response in the acute phase, thus interfering with the accuracy of prediction (36, 37). Therefore, subgroup analysis of stroke severity should be added to future studies.

The relationship between serum globulin and PSCI remains unclear, and studies on its correlation are limited. In the present study, serum globulin was combined with lymphocyte count for the first time to investigate the association between their ratio and PSCI. Elevated serum globulin has been associated with cancer, rheumatoid diseases, chronic liver disease, nephrotic syndrome, and diabetes mellitus (38). Long-term chronic inflammation is often accompanied by elevated serum globulin levels, indicating an overactive immune system (39). A previous study by Gao et al. found that serum globulin levels were elevated in AD patients and that there was a significant positive correlation between serum globulin levels and cerebrospinal fluid Aβ42 and its associated biomarkers, suggesting that serum globulin may be involved in the pathophysiologic mechanisms of AD (40). One study found that the association between serum globulin levels and cognitive impairment in older Americans was non-linear; higher serum globulin levels were associated with a higher risk of cognitive impairment (41). Another research suggests positive and non-linear correlations between the albumin-to-globulin ratio and cognitive function in older Americans (42). The results of this study showed that both univariate and multivariate logistic regression analyses of GLR were independently associated with PSCI. Upon further construction of the predictive model, our findings revealed that the OR (95% CI) for the highest tertile of GLR compared to the lowest tertile was OR = 6.20 (95% CI, 2.10–18.33; *p* = 0.001). Meanwhile, ROC curve analysis revealed that the AUC of GLR for predicting PSCI was 0.726, indicating its good predictive efficacy. Therefore, we believe that GLR is a promising hematological biomarker that should be further proven through additional prospective studies.

This study also found that patients in the PSCI group had significantly lower hemoglobin levels than those in the PSNCI group. A multicenter prospective cohort study found that the Hb level was negatively associated with the risk of PSCI within 3 months of stroke onset and was an independent protective predictor of PSCI (43). Another cohort study found that low Hb levels were associated with an increased risk of PSCI (44). Hb is the main protein that delivers oxygen to tissues throughout the body, and the brain accounts for 20% of the body’s oxygen consumption; low hemoglobin leads to cerebral hypoxia, which in turn decreases brain tissue metabolism and neuronal activity, further leading to mitochondrial dysfunction, oxidative stress, and inflammatory responses and ultimately cognitive dysfunction; low Hb-associated iron deficiencies in the brain may also affect neurotransmitter metabolism and function (44, 45). In contrast to the results of some previous studies, our findings showed that TC and LDL-C levels were significantly lower in patients in the PSCI group than in the PSNCI group (46–48). First, this difference may be caused by unbalanced comorbidities. For example, the proportion of patients with coronary artery disease was higher in the PSCI group, and the administration of lipid-lowering medications can lead to low baseline lipid levels. Second, previous studies have suggested that low cholesterol levels may be associated with poorer cognitive performance. The results from a prospective cohort study of 407,190 individuals showed that demented patients had lower TG and LDL levels than the non-demented population (49). Another cross-sectional study that included 1,754 individuals from a community-based population also showed that mildly elevated LDL-C levels were associated with better cognitive function (50). Plasma cholesterol promotes the structural integrity of nerve cells and regulates their mobility; cellular cholesterol deficiency or insufficient supply of cholesterol to neurons has been demonstrated to inhibit dendritic growth and synapse formation and to induce neurodegenerative pathologies (51). Cholesterol depletion may also increase the risk of blood–brain barrier rupture, leading to progressive synaptic and neuronal dysfunction and cognitive impairment (52).

In addition, our study found that patients in the PSCI group were older, consistent with previous findings (53, 54). A multicenter cross-sectional study in China found that the prevalence of PSCI increased with age, from 71.2% in 250 people aged 19–44 years to 84.2% in 8,076 people aged 75 years or older (55). The possible explanation for this is that the brains of older adults are less efficient at generating and operating compensatory mechanisms to mitigate the cognitive decline associated with stroke and aging (56). Meanwhile, the results of univariate logistic regression analyses indicated that education level independently influenced the occurrence of PSCI. The multivariable logistic regression analysis remained unchanged after adjusting for clinical variables (OR, 0.78; 95% CI, 0.68–0.90; *p* < 0.001). Higher levels of education were associated with a lower incidence of PSCI, consistent with previous studies (53, 55, 57). Higher levels of education indicate better cognitive reserve, which may lead to more compensatory neural resources to counteract age-related pathophysiologic changes (58).

## 5 Limitations

Our study has some limitations. First, our data came from a single center with a small sample size. Second, since PSCI may occur immediately after stroke and last for 6 months, and this study only assessed cognitive function in the acute post-stroke period, we lacked subsequent serial follow-up of patients. Third, post-stroke depression (PSD) is a prevalent complication following a stroke and is closely linked to PSCI. The clinical symptoms of PSD, such as memory decline, overlap with those of PSCI, potentially complicating the diagnosis of PSCI (59, 60). However, this study did not include depression screening for the subjects, which may affect the assessment of PSCI. Fourth, some patients who could not cooperate in completing the cognitive assessment (speech disorders, dysarthria, visual deficits, etc.) were excluded from our study, which would have caused some selection bias. Finally, the diagnostic accuracy of peripheral GLR for PSCI in the acute phase of stroke is moderate, and we look forward to further studies to explore its actual value.

## 6 Conclusion

We demonstrated for the first time that circulating GLR in the acute phase of stroke can serve as a predictive indicator of the occurrence of PSCI and has diagnostic value, providing a new perspective for clinical research on the peripheral immune-inflammatory response associated with PSCI. In particular, cognitive assessment in the acute phase of stroke was performed 7–10 days after stroke, during which cerebral infarction symptoms were relatively stable, and interference from cognitive stress injury caused by the stroke itself could be avoided. In addition, the predictors of our study are easy to obtain in the clinic, have low testing costs, and are easy to generalize.

## Data Availability

The raw data supporting the conclusions of this article will be made available by the authors, without undue reservation.
